# Shift work, cancer and "white-box" epidemiology: Association and causation

**DOI:** 10.1186/1742-5573-7-11

**Published:** 2010-11-30

**Authors:** Thomas C Erren

**Affiliations:** 1 Institute and Policlinic for Occupational Medicine, Environmental Medicine and Prevention Research; University of Cologne, Kerpener Straße 62, Haus 11 B, 50937 Köln, Germany

## Abstract

This commentary intends to instigate discussions about upcoming epidemiologic research, and its interpretation, into putative links between shift work, involving circadian disruption or chronodisruption [CD], and the development of internal cancers.

In 2007, the International Agency for Research on Cancer (IARC) convened an expert group to examine the carcinogenicity of shift work, inter alia characterized by light exposures at unusual times. After a critical review of published data, the following was stated: "There is *sufficient evidence *in experimental animals for the carcinogenicity of light during the daily dark period (biological night)". However, in view of limited epidemiological evidence, it was overall concluded: "Shiftwork that involves circadian disruption is *probably carcinogenic to humans (Group 2A)"*.

Remarkably, the scenario around shift work, CD and internal cancers provides a unique case for "white-box" epidemiology: Research at many levels - from sub-cellular biochemistry, to whole cells, to organs, to organisms, including animals and humans - has suggested a series of quite precise and partly related causal mechanisms. This is in stark contrast to instances of "black box" or "stabs in the dark" epidemiology where causal mechanisms are neither known nor hypothesized or only poorly defined. The overriding theme that an adequate chronobiological organization of physiology can be critical for the protection against cancer builds the cornerstone of biological plausibility in this case.

We can now benefit from biological plausibility in two ways: First, epidemiology should use biologically plausible insights into putative chains of causation between shift work and cancer to design future investigations. Second, when significant new data were to become available in coming years, IARC will re-evaluate cancer hazards associated with shift work. Biological plausibility may then be a key viewpoint to consider and, ultimately, to decide whether (or not) to pass from statistical associations, possibly detected in observational studies by then, to a verdict of causation.

In the meantime, biological plausibility should not be invoked to facilitate publication of epidemiological research of inappropriate quality. Specific recommendations as to how to design, report and interpret epidemiological research into biologically plausible links between shift work and cancer are provided.

Epidemiology is certainly a poor tool

for learning about the mechanism

by which a disease is produced,

but it has the tremendous advantage

that it focuses on the diseases and the deaths

that actually occur,

and experience has shown that it continues to be second to none as

a means of discovering links

in the chain of causation

that are capable of being broken.

-Sir Richard Doll [1]

## Introduction

An editorial [2] accompanying a 2010 special theme issue of the *Scandinavian Journal of Work, Environment & Health *pointed out that shift work has been a public health concern since the 1800s. Understandably, a large number of studies have addressed numerous health concerns associated with shift work. However, only recently has a new candidate effect been added to the list, namely cancer.

With the following, I would like to instigate and fuel discussion of challenges which inevitable shift work, and research into shift work and cancer, will pose in the future. First, I provide background to understand how the International Agency for Research on Cancer [IARC] has provided the incentive for epidemiological studies which investigate "probable" cancer hazards associated with shift work [3]. Second, I explain why researching the 'shift work-cancer-conundrum' provides a unique case for "white-box" epidemiology. Third, I suggest how to design, report and interpret observational investigations into biologically plausible links between shift work and cancer.

## Discussion

A key reason why coming years will bring a cascade of epidemiologic studies into putative links between shift work and cancer has been provided in 2007: At that time, IARC convened an expert group to examine the carcinogenicity of shift work, inter alia characterized by light exposures at unusual times. After a critical review of published data relevant to an assessment of carcinogenicity, the following was stated: (i) "There is *sufficient evidence *in experimental animals for the carcinogenicity of light during the daily dark period (biological night)". (ii) "There is *limited evidence *in humans for the carcinogenicity of shiftwork that involves night work". On the basis of (i) and (ii), the 22 working group participants concluded, (iii) "Shiftwork that involves circadian disruption is *probably carcinogenic to humans (Group 2A)"*.

Importantly, the panel judged the evidence from studies of cancer in experimental animals, and from mechanistic and other relevant data, to be so strong that even a Group 1 classification (*carcinogenic to humans*) of shift work was contemplated. At the time, though, the epidemiological evidence in humans for the alleged links was considered limited, thus the Group 2A classification (*probably carcinogenic to humans*) on balance of all scientific literature that was openly available, i.e., published or accepted for publication, in 2007.

One future scenario to place shift work, involving circadian disruption or chronodisruption, in the Group 1 category would be when evidence of carcinogenicity in humans were no longer *less than sufficient *as it was considered to be in 2007 [3]. With the given background, it seems not unrealistic to anticipate the following: Provided that we are going to see a number of 'positive association' studies in coming years, the "probable" link between shift work, circadian disruption and cancers in humans can likely be judged as being causal. To clarify, 'positive association' studies are meant to denote studies in which we were to observe that shift work, or some facet of this organizational feature of work, is positively associated with cancer. In other words, 'positive association' studies would be investigations showing a higher cancer rate in cohorts who are exposed than in cohorts who are not exposed to shift work or to detect a higher likelihood of exposure to shift work in cancer cases than in controls without the disease.

In 1965, Bradford Hill [4] suggested a set of "nine different viewpoints from all of which we should study association before we cry causation". In the case around shift work and cancer it seems to me that the state-of-affairs may be different, i.e., somewhat shortened and to be possibly settled on one condition: Indeed, one likely line of action might be a Group 1 classification of shift work with circadian disruption under the surmised scenario of 'positive association' studies in the next years. Put differently: If there were epidemiological reports of positive statistical associations in the near(er) future, the question might not be "association or causation" [4]. Rather, we may be facing the following equation: 'Association = causation'. In simple, albeit provocative, words, 'bring us the associations and we will call it causation'.

### Shift work, cancer and epidemiology

The background facts are bleak: On the one hand, shift work is inevitable today as there is work to do for people over 24 hours, everyday. To deliver such work at unusual times, 15-20 percent of male and female workers in Europe and the USA are engaged in regimens involving night work, i.e., in time-windows that contain the so-called biological night [3]. On the other hand, possibly associated breast and prostate cancers are two of the most common cancers in the world. Given the fact that many shift workers may be exposed to circadian disruption or chronodisruption, even small - let alone substantial - risk elevations could translate into numerous attributable cases. Therefore, resolution of the question whether we are looking at a chain of causation between shift work, CD and the development of cancer is very important for public health. That epidemiologists will have a key role in the upcoming quest for associations seems reasonable to expect. In line with Doll's 1996 statement quoted earlier, observational studies will now provide the means to look with care for the causes of cancers which actually occur in female and male shift workers.

### The case for "white-box" epidemiology

Conceptually, the 'shift work-cancer-conundrum' confronts us with a unique situation of "white-box" epidemiology [5]: A series of - quite precise and partly related - causal mechanisms has been suggested. Empirically, several biological mechanisms [[3]: "Exposure to light at night disturbs the circadian system with alterations of sleep-activity patterns, suppression of melatonin production, and deregulation of circadian genes involved in cancer-related pathways"] have been established in animal experiments and by mechanistic and other data. In addition, a generalized "chronodisruption-cancer-theory" [6] was proposed. Taken together, advanced biological knowledge, namely experimental insights into biologically plausible disease mechanisms involving circadian disruption or chronodisruption, can provide the basis for coherent "white-box" epidemiology. This is in stark contrast to instances of "black box" or "stabs in the dark" [7] epidemiology: There, at least when the studies are designed and analyzed, causal mechanisms are neither known nor hypothesized or only poorly defined. In the case of shift work and cancer, biological insights and plausibility can now be used at two critical stages of the scientific and public health endeavour: To appropriately collect, and to ultimately weigh, observational evidence in the course of the causal inference process. In short, abundant biomedical insights should be used to design and refine observational studies soon and can contribute to weighing observed evidence for and against causality later.

So, how can "white-box" epidemiology contribute to collecting observational data? To this end, "Building on laboratory findings may allow epidemiologists to examine more specific forms of exposure, disease, and their relation, which leads to more rigorous testing. When the biological context can be used to improve measurement of dose or specify modifiers of the association, the resulting epidemiologic study is enhanced" [7]. By incorporating insights into biology in their design, epidemiological studies may capture associations as expressions of biological cause-effect-relationships that are at work (or not) more precisely. In principle, "white-box" epidemiological studies can produce higher, lower or zero risks when compared with results observed in studies which were designed without detailed mechanistic knowledge.

And how can "white-box" epidemiology contribute to ultimately weighing the overall evidence for or against causality? A lucid way to illustrate the process of causal inference may be borrowed, albeit with variation, from Raymond Neutra: "Indeed a heuristic that is used for deciding if an agent is hazardous is that a "good story" can be told how the agent acts at each level of physical, chemical and biological organization" [8]. Usually, "the "story" is pieced together from many experiments [and observations] over time. Under this heuristic, the more steps on the story one can fill in, the more one believes that the link between [shift work and cancer] .... is causal in nature" [8]. But in the context of this paper, beyond contributing to telling a "good story" [8], "white-box" epidemiology can be a means to answer key questions: 'How good is the story?' and, of paramount interest, 'how true is the story for public health?' Clearly, it is conceivable that scientific reasoning and data evince an aesthetic thread. Indeed, we can think of scenarios where numerous steps are filled in and make a "story" [8] (seemingly) good to hear. But, equally clearly, "white-box" epidemiology can put all the animal experiments, mechanistic and other data in their proper community and medical perspective.

In theory, therefore, "white-box" epidemiology can contribute to lending (or not) credibility to the story about shift work and cancer. That story appears already pretty good in IARC's view. Yet, "white-box" epidemiology can now be critical to tell us whether (or not) the story is wrong or 'likely true' for humans. With regard to identifying 'true positives', the 'likely' carries a dual qualification: First, insofar as observational research will always disallow to verify that, what experiments would predict, is valid. Recall Popper's swan example: Even observing one million white ones would not be proof that all swans are white. However, observing just one black swan will do to falsify the 'all-swans-are-white hypothesis' [9]. Second, even the best story may still turn out, despite all inferential scrutiny, as a 'false positive'. After all, causal inference will remain a judgement, not a certainty. No set of studies can produce 100 per cent certainty that associations reflect a truly causal effect. Quite differently, with regard to 'true negatives', straightforward failure of "white-box" epidemiology to observe what experimental animals, mechanistic and other relevant data predict would actually render the "story" [8] untrue.

In practice, let us suppose now that studies, designed on the basis of biological or mechanistic insights, were to show zero risks. On the one hand, reliable studies with null results could imply that some shift work conditions may be more favourable than others. This could offer much-needed information for IARC under which circumstances shift work may cause cancer and under which not. On the other hand, flat indications of zero cancer risks in biologically targeted epidemiological studies could be real-life observations signalling that - despite being suggested by experimental data - the links-in-question may be entirely irrelevant for humans. Such demonstration of "*Evidence suggesting lack of carcinogenicity" *[[10]; p. 20] in epidemiological studies should actually rule out an IARC Group 1 classification.

Let us suppose this time that epidemiologic studies, refined by biological insights, were to convincingly report statistical associations between shift work, CD and cancers in man. We could derive several benefits from such epidemiological associations. As one benefit, they would tell us that a proposed "biochemical mechanism may well be at work in the real world" [11]. As another benefit, and as a consequence for public health, biological mechanisms derived from experimental animals, and from mechanistic and other relevant data, could provide the biological rationale to interpret statistical associations ultimately as reflecting a cause and effect phenomenon. Shift work would - under the surmised scenario - most likely be classified as a Group 1 carcinogen, i.e., it "*is carcinogenic to humans*" [10]. To fully appreciate the important role of biological plausibility for the International Agency for Research on Cancer, please note also the following.

Suppose in this final scenario that IARC experts were to judge evidence of carcinogenicity in humans to be less than sufficient, for example, limited as in 2007. In such case, however, IARC's door is still open to put the Group 1 label to an agent, or to an organizational work-place feature like shift work, on the basis of biological insights and plausibility alone. To do that, there must be sufficient experimental animal evidence and strong mechanistic evidence in exposed humans that the agent acts via a relevant route that leads to cancer. This is evinced when one reads the preamble to "IARC Monographs on the Evaluation of Carcinogenic Risks to Humans" [[10]; p. 22].

### IARC Group 1: The agent is *carcinogenic to humans*

"This category is used when there is *sufficient evidence of carcinogenicity *in humans. Exceptionally, an agent may be placed in this category when evidence of carcinogenicity in humans is less than *sufficient *but there is *sufficient evidence of carcinogenicity *in experimental animals and strong evidence in exposed humans that the agent acts through a relevant mechanism of carcinogenicity".

### The imperative to have quality studies

In his 1994 commentary [7], from which I have quoted already twice, David Savitz wrote: "With sufficient understanding of the disease process based on disciplines other than epidemiology, there may be little need for epidemiology to guide clinical practice or policy. Epidemiology of limited quality or quantity may suffice, concentrating resources on the situations in which refinements of epidemiologic information will be most beneficial". Now, in my view, Dr. Savitz' premise "With sufficient understanding of the disease process based on disciplines other than epidemiology" is fulfilled in the case of the hypothesized links between shift work, CD and the development of internal cancers. But, importantly, with the sequitur "Epidemiology of limited quality or quantity may suffice" I can not agree. To clarify my point: A key requirement for any published study must be the quality and validity of the study results and we need, of course, appropriate replication ("quantity") of epidemiological findings. Granted, we are looking at abundant biomedical insights in disease processes from disciplines beyond epidemiology, as is evinced by the 2007 IARC conclusion of "sufficient evidence in experimental animals for the carcinogenicity of light during the daily dark period (biological night)" [3]. This should, however, not confer upon us the freedom to be more flexible with regard to the quality and replication standards of observational studies in this field.

A key requirement, and key criterion, for publication must be the quality of the studies. That biological plausibility may be invoked to facilitate publication of epidemiological research of inappropriate quality must be avoided.

### What may qualitative and quantitative reviews tell us about the putative nexus of shift work, circadian disruption or chronodisruption and cancer?

One way to systematically pave the road to quality studies is that we try to understand what studies regarding shift work and cancer have already been conducted and how. One means to exploit what is already there before planning new observational studies is to conduct qualitative and quantitative reviews, including meta-analyses as a "study of studies" [12].

When reviewing the scientific literature, it should certainly surprise that, so far, "circadian disruption" as the critical link in the postulated chain of causation between shift work and cancer has not been defined. Indeed, the questions 'what is circadian disruption?' and 'how can we sensibly measure circadian disruption in observational studies?' are still unanswered. In contrast, the phenomenon of chronodisruption, which was operationalized in a qualitative and quantitative review [13] summarized below and led independently of IARC to very similar results, was first described in 2003 [14] and systematically defined in 2009 [15].

Chronodisruption [CD], it was suggested [13-15], is a relevant disturbance of the circadian organization of physiology, endocrinology, metabolism and behaviour, which links light, biological rhythms and the development of cancers. More specifically, in 2008, the possible links between CD and the development of cancers were extended within a generalized theory [6]. "This theory holds that CD can be understood as a critical loss of time order, i.e. a disorder or chaos of an otherwise physiological timing at different organizational levels, including the gene expression levels in individual cells" [15]. And it was further specified that CD be "a breakdown of phasing internal biological systems appropriately relative to the external, i.e. environmental changes, which leads to chronobiological disorders" [15]. One long-term consequence of this chronodisruption is presumed to be the development of cancer. Importantly, "while melatonin as a key time messenger and time keeper can be a marker of CD, it is probably only partially related to the differential cancer occurrence apparent in individuals who chronically or frequently experience an excess or deficit of chronodisruption" [6].

30 epidemiological studies were found eligible for meta-analyses of breast or prostate cancer risks in flight and shift personnel who were considered to have been exposed to chronodisruption. Interestingly, statistical analyses did not provide specific warning against combining data in subsets of the 30 included studies. A 70% and 40% increase in the risk of breast cancer in flight personnel and shift personnel, respectively, and excess relative risks of prostate cancer in flight personnel were calculated. Moreover, the two studies of prostate cancer in male shift workers available at the time were both compatible with increased risks [16,17]. And yet, with regard to the observed increased risks in shift workers and flight personnel the following was concluded: "in view of doubts about whether the differing assessments of CD can really be regarded as valid reflections of the same causative phenomenon and the lack of control of covariates in the majority of studies, it is premature to conclude that the risk observations reflect a real, rather than spurious, association with CD. The challenge for future epidemiological investigations of the biologically plausible links between chronodisruption and human cancers is to conduct studies which appreciate details of transmeridian travelling, of shift work and of covariates for the development of the diseases" [13].

On the basis of the qualitative and quantitative review, a series of recommendations for future epidemiological studies was developed. Apart from standard information in cancer research such as the workers' ages, medication, exercise et cetera, future epidemiologic studies must include critical details of shift work: The length of the shift, the speed of changes in schedules, and the direction, i.e., forward or clockwise and backward or counter-clockwise rotating shifts [18]...and so on.

As another consequence of the 2008 review, in order to assess chronodisruption approriately, so-called chronodisruptors have been defined [15]. This was done to identify facets which epidemiologists might want to consider when doing their studies in the shift work and cancer field. In addition to much-needed details on the shift work exposures [19], it is here that we should be as careful as possible when planning, conducting, analysing and interpreting epidemiologic studies: "Chronodisruptors are exogenous and endogenous exposures or effectors which are chronobiolocially active and can thus disrupt the timing and order, i.e. temporal organization of physiologic functions and hierarchies. In principle, whatever allows the establishment of temporal organizational order in organisms should also be capable of disrupting such order or temporal programme when present or applied in excess or deficit and, most importantly, at unusual and inappropriate times, especially if combined with further agonistic or antagonistic chronobiologial effectors" [15]. In view of the *Zeitgeber *multiplicity to which shift workers are exposed [20], any observational study should include information on, and/or discussion of, the following intermediates or covariates, and their possible interactions, along the postulated chain(s) of cancer causation: Light, melatonin, sleep, food [21-23], work and leisure activities, biological stress and ambient noise [20].

A prime variable identified for future studies, not included in the observational studies published until 2008 [13], and indeed until today, would be the chronotype of study individuals. Note, that so-called 'owls' are likely to suffer considerably less from nightshift work than the 'lark-types' in human populations. Ultimately, therefore, the chronotype may explain why among workers, who share the same shift work exposures, some may develop cancer and others not or why some develop it earlier and others later. In addition, it seems reasonable to consider chronotype also in studies which contrast groups with assumed major differences in their average exposures to shift work. In such studies, individuals' chronotype could be a covariate which might help to explain within-group variations of cancer. Yet, there is one more reason as to why adjusting for chronotype characteristics could critically improve observational studies: Such information can be obtained and interpreted more readily than information on clock gene variants or polymorphisms [24] and other genetic characteristics in sizeable populations. Intriguingly, from a mechanistic point of view, the chronotype may actually mirror genetic information which is otherwise difficult to include and consider.

### What epidemiological studies could be conducted in a near(er) future: a sobering look ahead

The need to resolve biologically plausible cancer hazards associated with shift work will lead to a host of observational studies into the issues involved. Each epidemiological study will provide information in its own right. In addition, such studies as a whole may bring us the significant new data which can prompt a re-evaluation by IARC of what exactly is the nature of links between shift work, CD and internal cancers.

Disconcertingly, there are two reasons that with some probability we will witness - possibly erroneously - 'positive association' studies in the nearer future. First, publication bias still facilitates publication of 'positive studies' [25]. This is particularly true in a research field such as shift work and cancer which does already attract considerable public interest. Second, case-control studies which obtain exposure information via questionnaires rather than being based on (rather) unbiased industry-records [26] can be conducted in a relatively fast fashion. However, such studies may be prone to critical self-selection bias among the controls and to recall bias among the cases. This problem of falsely producing 'positive risk estimates' was recently highlighted by Pirie and colleagues. Their qualitative and quantitative review showed that, in some studies of passive smoking and breast cancer, women were more likely to report past exposures because they knew that they had developed breast cancer [27].

## Conclusions

Overall, I can't put my conclusions too strongly. Experimental researchers from many disciplines are working at every level to understand the nature of the temporal organisation of biology - from sub-cellular biochemistry, to whole cells, to organs, to organisms, including animals and humans - and its implications for the development of cancer. And yet, it will be up to epidemiology to provide, in line with Sir Richard Doll's quote prefacing this text, key observational evidence as to whether, what appears biologically plausible today, has relevance for public health. Necessary real-life studies will be a challenge [28], but epidemiologic investigations of the suspected links and causal interpretation of possibly observed associations should benefit from 'boxes' which are not black and empty [29] but rather white and full with plausible disease mechanisms provided by experimental scientists.

Importantly, though, we must be prepared for and remain critical of qualitatively inferior studies in a nearer future as they may invoke the suggestive biological plausibility to support, and get published, invalid epidemiologic results.

In the meantime, I think that another reference to Hill's seminal paper [4] is warranted with regard to possible prevention: "All scientific work is incomplete - whether it be observational or experimental. All scientific work is liable to be upset or modified by advancing knowledge. That does not confer upon us a freedom to ignore the knowledge we already have or to postpone the action that it appears to demand at a given time (p 300)" [4]. In this vein, syntheses of our current knowledge on countermeasures towards adverse effects of shift work [30-32], are welcome. In addition, the frequent evaluation 'when to act and when to study' should be modified in the complex fields of shift work and health. More generally, we do need both, studies and actions with regard to generating shift work conditions with less or, preferably, no impact on cancer developments and other detrimental health outcomes. More specifically, despite numerous shift work investigations to-date, we need to study further *how *to act. In the latter respect, reliable studies with null results must be published [33]: They may not only be important to the overall assessment as to whether shift work conditions lead to increased cancer risks in some individuals but also that some shift work conditions may be more favourable than others. Provided that associations between CD and cancer were eventually to be interpreted as causal, real life observational studies would be a basis for assessing what shift work exposures, and what level of CD, may be tolerable and what is unacceptable.

To foster "white-box" epidemiological studies and to ultimately reach sound public health decisions regarding shift work and cancer, here are seven recommendations:

### How biological plausibility should be used to design, to report and to interpret epidemiological studies

1. Biological insights and plausibility should be used to design and refine epidemiologic investigations. The challenge for studies of biologically plausible links between shift work, CD and human cancers is to appreciate details of shift work and of covariates for the development of the diseases.

2. We shall need observational studies of different shift-regimens at different workplaces. Moreover, we will need such studies for women and for men. Such studies should be - whenever possible - adjusted for chronotype information. This can be obtained and interpreted more readily than information on clock gene variants [24] and other genetic characteristics in sizeable populations. In addition, such chronotype information may encompass a lot of other information relevant to study individuals' chronobiology in a feasible fashion.

3. In view of the wealth of biological information and seemingly overwhelming evidence in support of mechanistic causality, the primary objective of the studies should be to accurately collect and present methods and data. Rather than over-interpretation of observed associations as reflecting causal relationships, this very focus on epidemiological facts could allow "writers more time and the report more space to describe the methods, analyses and data" [34]. Authors should let peers reading detailed descriptive accounts of possibly important epidemiological research develop explanations for the findings.

4. Key criterion for publication must be the quality of the studies. Biological plausibility should not be invoked to facilitate publication of epidemiological research of inappropriate quality.

5. The outcome of 'biological plausibility' should be considered open. We must not be hypnotized by the tantalizing status quo of experimental and mechanistic evidence. Indeed, we should be prepared to decide ourselves, or be guided by more experts, on the evidence available in coming years. For instance, new animal data then may make strongly incriminating mechanistic evidence available today less or no longer persuasive. Thus, how the available experimental evidence has to be judged then, whether it provides - still, less or even more so - a biological rationale to guide the design of observational studies and to interpret observations as reflecting (or not) a cause-effect-phenomenon, is open today.

6. The outcome of "white-box" epidemiological studies should be considered open. Making use of insights into biological candidate mechanisms may lead to (very) different studies with (very) different results, both with regard to the direction and the magnitude of putative cancer risks. In the future, biologically targeted studies may show (much) higher, lower or no risks at all.

7. Ultimately, biological insights and plausibility should be used to weigh the overall scientific evidence to rule in or rule out causality. Insights into plausible mechanisms may then provide a biological rationale to judge that associations observed between shift work and cancer do (or do not) reflect a cause and effect phenomenon.

## Competing interests

The author declares that they have no competing interests.
